# Multiomics integration reveals the effect of Orexin A on glioblastoma

**DOI:** 10.3389/fphar.2023.1096159

**Published:** 2023-01-20

**Authors:** Sha Yang, Renzheng Huan, Jianhe Yue, Jin Guo, Mei Deng, Liya Wang, Shuo Peng, Xin Lin, Lin Liu, Jia Wang, Guoqiang Han, Yan Zha, Jian Liu, Jiqin Zhang, Ying Tan

**Affiliations:** ^1^ Guizhou University Medical College, Guiyang, Guizhou Province, China; ^2^ Department of Neurosurgery, The Second Affiliated Hospital of Chongqing Medical University, Chongqing, China; ^3^ Department of Neurosurgery, Guizhou Provincial People’s Hospital, Guiyang, China; ^4^ Department of Nephrology, Guizhou Provincial People’s Hospital, Guiyang, China; ^5^ Department of Respiratory and Critical Care Medicine, Guizhou Provincial People’s Hospital, Guiyang, China; ^6^ Department of Neurosurgery, Chongqing Emergency Medical Center, Chongqing, China; ^7^ Department of Anesthesiology, Guizhou Provincial People’s Hospital, Guiyang, China

**Keywords:** glioblastoma multiforme, Orexin A, multi-omics, functional enrichment analyses, genes

## Abstract

**Objectives:** This study involved a multi-omics analysis of glioblastoma (GBM) samples to elaborate the potential mechanism of drug treatment.

**Methods:** The GBM cells treated with or without orexin A were acquired from sequencing analysis. Differentially expressed genes/proteins/metabolites (DEGs/ DEPs/ DEMs) were screened. Next, combination analyses were conducted to investigate the common pathways and correlations between the two groups. Lastly, transcriptome-proteome-metabolome association analysis was carried out to determine the common pathways, and the genes in these pathways were analyzed through Kaplan-Meier (K-M) survival analysis in public databases. Cell and animal experiments were performed to investigate the anti-glioma activity of orexin A.

**Results:** A total of 1,527 DEGs, 52 DEPs, and 153 DEMs were found. Moreover, the combination analyses revealed that 6, 4, and 1 common pathways were present in the transcriptome-proteome, proteome-metabolome, and transcriptome-metabolome, respectively. Certain correlations were observed between the two data sets. Finally, 11 common pathways were discovered in association analysis, and 138 common genes were screened out in these common pathways. Six genes showed significant differences in terms of survival in both TCGA and CGGA. In addition, orexin A inhibited the proliferation, migration, and invasion of glioma *in vitro* and *in vivo*.

**Conclusion:** Eleven common KEGG pathways with six common genes were found among different omics participations, revealing the underlying mechanisms in different omics and providing theoretical basis and reference for multi-omics research on drug treatment.

## 1 Introduction

Glioblastoma (GBM) is the most common malignant central nervous system (CNS) tumor in adults, and this tumor is very aggressive (Batash et al., 2017; Louis et al., 2021). Even after surgery, radiotherapy, and chemotherapy, the median overall survival (OS) of patients with GBM is approximately 15 months (Johnson et al., 2018; Witthayanuwat et al., 2018). Tumors may continue to grow (progress) and accompany by recurrence after treatment (Weller et al., 2017; Bates et al., 2018; Tan et al., 2018). Although some advances have been realized in the treatment of GBM, such as the application of temozolomide and bevacizumab, patients with GBM still encounter poor treatment effect and drug resistance. (Tan et al., 2017; Jiapaer et al., 2018; Kickingereder et al., 2020; Reardon et al., 2020; Nayak et al., 2021; Tesileanu et al., 2022; Zeng et al., 2022). Tumor therapeutic field (TTF) therapy has also been approved by the FDA for recurrent (2011) and newly diagnosed (2015) GBM, but the lack of generalizability of data from previous studies has prevented TTF from being widely used. (Regev et al., 2021). Therefore, no effective treatment is currently available for GBM, which remains one of the most difficult and complex cancers to treat. Therefore, understanding the complex mechanism of GBM and exploring new therapeutic strategies are urgently required.

Orexin is involved in the interaction between cancer and neurodegenerative diseases such as narcolepsy. (Liu et al., 2015; Boss and Roch, 2017; Wan et al., 2017; Tan et al., 2020). Orexin A and B (OXA and OXB), also known as hypocretin 1 and 2, respectively, are peptides expressed by hypothalamic neurons and are first identified in 1988. (Sakurai et al., 1998). Orexin binds to two G-protein-coupled receptors, namely, orexin receptors 1 and 2 (OR1 and OR2) (Sakurai et al., 1999), which are widely expressed in the CNS (de Lecea et al., 1998), and is consistent with widespread expression of orexin neurons. (Tsujino and Sakurai, 2009). Orexin signaling is multifaceted and complex, with similar mechanisms for OR1 and OR2. A recent *in vivo* study reported that OXA may increase mitophagy and disrupt mitochondrial structure. (Zhu et al., 2021). In addition, OXA may affect the interaction between brain and gastrointestinal tract by acting on intestinal microorganisms, thereby affecting brain function (Foster et al., 2016). In recent years, a series of studies has focused on modulating orexin-related signaling, which may play a surprising therapeutic role in the treatment of certain types of cancer (Graybill and Weissig, 2017; Mogavero et al., 2021). OXA stimulates neovascularization, a key step in chronic inflammation and tumor growth (Kim et al., 2015). OR1 signaling has a pro-apoptotic role signaling in neuroblastoma cell lines (Rouet-Benzineb et al., 2004). These results were later confirmed in other tumor cells both *in vivo* and *in vitro* (Voisin et al., 2011; Wen et al., 2016). In this process, OXA directly activates caspase-3 to promote tumor cell apoptosis *in vivo*, or this process is possibly mediated by two immunoreceptor tyrosine-based inhibitory motifs in OR1 and OR2, which participate in phosphotyrosine phosphatase SHP2 and induce mitochondrial apoptosis (Mogavero et al., 2021). Studies on the potential modulation of the orexin system in cancer are still pioneering. Although promising results have been obtained, further human data are needed. Therefore, a multi-omics analysis was conducted based on transcriptome, proteome, and metabolome.

In recent years, molecular biology technology has been developed rapidly. Meta-analysis of single-omics datasets is very valuable for biological and medical research (Ni et al., 2014; Hanna et al., 2017; Goveia et al., 2020; Rijkschroeff et al., 2020; Hoogstrate et al., 2022). With the continuous development of deep sequencing and other high-throughput methods, the gradual reduction of cost and the increasing maturity of technology, a large number of omics data can be obtained, thus allowing the measurement of multi-omics data. Multi-omics analysis can reduce the effect of biological and experimental bias in the data, and different omics can reveal different cellular aspects, such as the effects manifested at the genomic and proteomic levels (Subramanian et al., 2020; Zhou et al., 2021). Multi-omics analysis is a comprehensive assessment of multiple sets of characteristics. Specifically, transcriptomics is the study of expressed RNAs; it usually focuses on protein-coding RNA (mRNA) and includes non-coding RNA that coordinate and regulate gene expression, which provides attention to the underlying mechanisms involved in genes and biological processes (de Jong and Bosco, 2021). Proteomics is the study of expressed proteins and is used to describe protein abundance, properties, post-translational modifications, and protein interactions (Aslam et al., 2017). Metabolomics focuses on the analysis of small molecules (i.e., metabolites), including carbohydrates, fatty acids, amino acids, and other compounds (Patti et al., 2012). Transcriptomics, proteomics, and metabolomics are complementary and synergetic for the effective understanding of molecular interactions and disease mechanisms.

In the present study, orexin-induced changes in GBM cells were analyzed at the transcriptomic, proteomic, and metabolomics levels by using a multi-omics approach. We measured multiple omics data, such as transcriptomics, proteomics, and metabolomics, of GBM cells cultured with and without Orexin A, and identified differentially expressed molecules that were significantly altered in each omics layer in orexin-added GBM cells relative to those without OXA, and linked these layers to differential regulation. The correlation of differential molecules was analyzed. Simultaneously, the KEGG pathways enriched by the combination of pairwise omics and the three omics were analyzed, and the differentially expressed molecules and differential regulators in KEGG pathways were extracted. Finally, data from TCGA and CGGA public databases were combined to screen out the molecules with prognostic effect. The underlying mechanism of OXA action in GBM cells was explored.

## 2 Results

### 2.1 Identification of DEGs, DEPs, and DEMs

**FIGURE 1:**
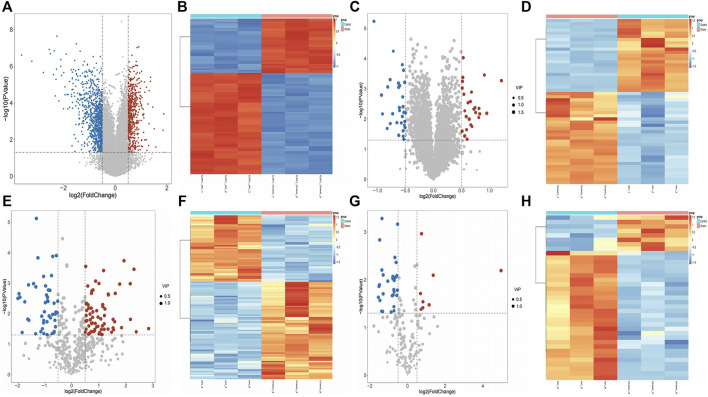
Differential expression analysis between case and control samples. **(A)** Volcano plots of differentially expressed genes (DEGs) between case and control samples with |log_2_fold change (FC)| > 0.5 and *p*-value < 0.05. **(B)** Heat map plots of the top 100 DEGs. **(C)** Volcano plots of differentially expressed proteins (DEPs) with |log_2_FC| > 0.5, *p*-value< 0.05, and VIP>1. **(D)** Heat map plots of the top 100 DEPs. **(E–F)** Volcano plot and heatmap of differentially expressed metabolites (DEMs) with |log_2_FC| > 0.5, *p*-value< 0.05, and VIP>1 in positive mode. **(G–H)** Volcano plot and heatmap of DEMs with |log2FC| > 0.5, *p*-value< 0.05 in negative mode. In the volcano plots, the red points represent upregulated genes, proteins, and metabolites, while the blue points represent downregulated genes, proteins, and metabolites. In heat map plots, the red point represents upregulated molecules, while the blue point represents downregulated molecules.

The differential expression analysis results illustrated that 1,527 DEGs were found between case and control samples, including 574 upregulated and 953 downregulated genes (Figures 1A, B). Moreover, 52 DEPs were screened out from the proteomic data, including 23 upregulated and 29 downregulated proteins (Figures 1C, D). In addition, in the positive mode of the metabolome data, 69 upregulated and 47 downregulated DEMs were identified from 481 metabolites (Figures 1E, F), and the results of 196 metabolites in negative mode showed 8 upregulated and 29 downregulated DEMs (Figures 1G, H). In total, 153 DEMs were identified (116 DEMs in positive mode, 37 in negative mode).

### 2.2 Functional enrichment analyses of DEGs, DEPs, and DEMs

**FIGURE 2:**
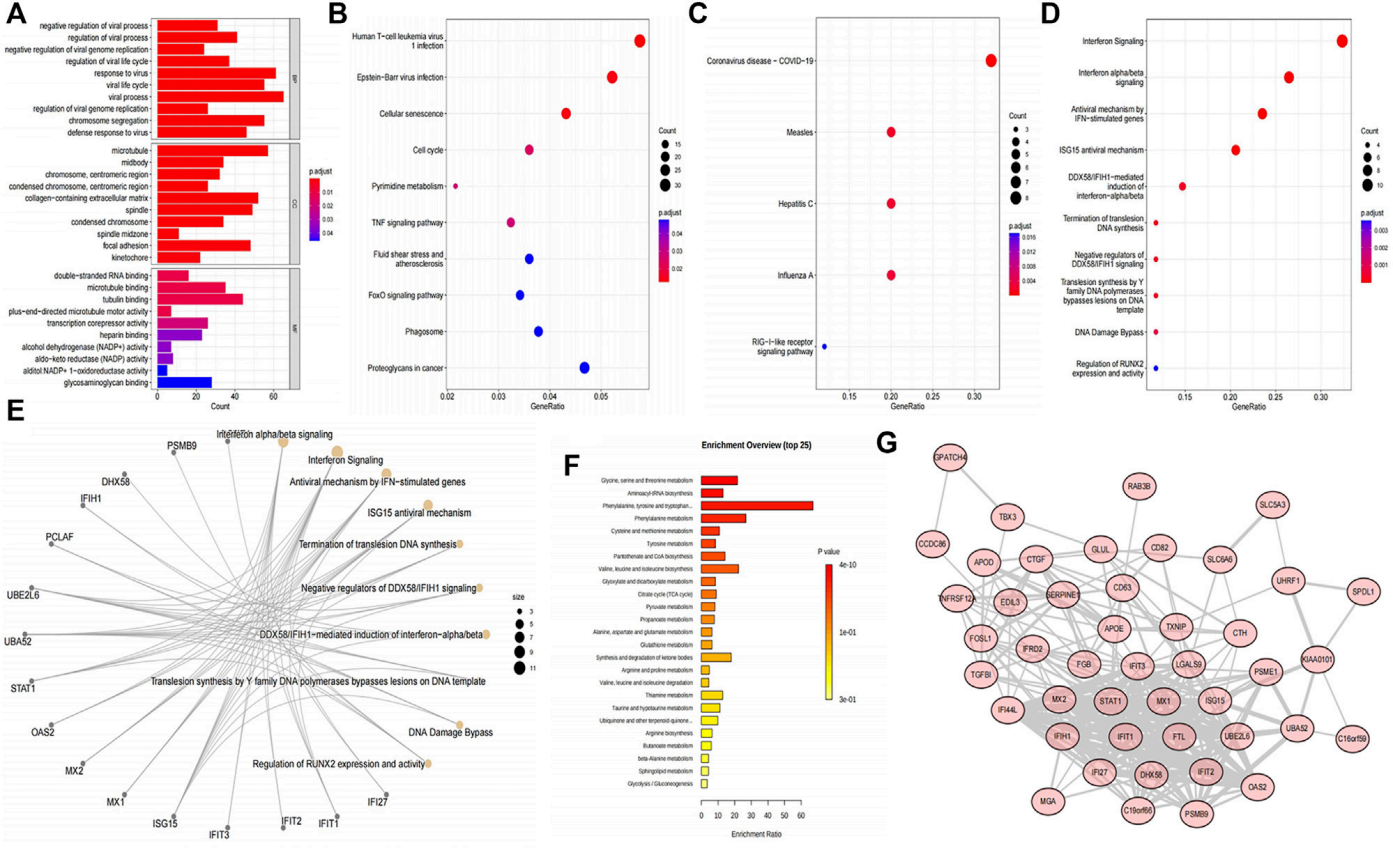
Functional enrichment analyses and construction of the protein-protein interaction (PPI) network. **(A)** Bar chart of the top 10 most enriched GO terms of the DEGs. **(B)** Bubble chart of the 10 most activated KEGG pathways of the DEGs. **(C)** Bubble chart of the five most activated KEGG pathways of the DEPs. **(D)** Bubble chart of the 10 most enriched REACTOME annotations of the DEPs. **(E)** Interaction network of the top10 REACTOME pathways and DEPs. Yellow nodes indicate the REACTOME pathways, gray nodes indicate the DEPs, and the lines indicate the interaction of proteins and the pathways. **(F)** Top 25 most activated pathways of the DEMs in MetaboAnalyst website. **(G)** PPI network of DEPs. The nodes represent the proteins, the lines represent the interaction relationship, and the line thickness was positively correlated with the combined score.

A total of 11 pathways and 806 GO terms were enriched by DEGs, including negative regulation of viral process, regulation of viral process, viral process, and response to virus in biological process (BP), midbody, microtubule, and collagen−containing extracellular matrix in cellular component (CC), tubulin binding, microtubule binding, and double−stranded RNA binding in molecular function (MF), and the Human T Cell leukemia virus 1 infection, Epstein-Barr virus infection, and cellular senescence in KEGG pathway. The diagrams show the top 10 enriched terms (Supplementary Tables S1A,B; Figures 2A, B). Moreover, the DEPs enriched five KEGG pathways and 136 REACTOME pathways, including the RIG−I−like receptor signaling pathway, influenza A, hepatitis C, measles, coronavirus disease—COVID−19 in KEGG and interferon signaling, interferon alpha/beta signaling, antiviral mechanism by IFN−stimulated genes, and ISG15 antiviral mechanism in REACTOME (Supplementary Tables S1C,D; Figures 2C, D). The top 10 REACTOME pathways were visualized into a network diagram (Figure 2E). Fourteen pathways were enriched by DEMs, such as glycine, serine, and threonine metabolism, aminoacyl-tRNA biosynthesis, and phenylalanine metabolism (Supplementary Table S1E, Figure 2F). In addition, in the protein-protein interaction (PPI) network of DEPs discovered that among 52 DEPs, seven discrete proteins were found, and the interaction network of the 45 remaining proteins was obtained, in which 45 nodes and 242 edges were observed. Therefore, these 45 intersection targets had strong interactions (Figure 2G). Notably, no common enriched KEGG pathway was observed among DEGs, DEPs, and DEMs. Therefore, the combined KEGG analysis cannot be performed.

### 2.3 Correlations between transcriptome-proteome, transcriptome-metabolome, and proteome-metabolome

Six common pathways were identified in the transcriptome-proteome group, including proteoglycans in cancer, Kaposi sarcoma-associated herpesvirus infection, chemical carcinogenesis—reactive oxygen species (ROS), coronavirus disease (COVID-19), mitophagy—animal, and p53 signaling pathway (Supplementary Table S2A, Supplementary Figure S1). Additionally, four common pathways were selected between transcriptome and metabolome data, such as pyrimidine metabolism, FoxO signaling pathway, PI3K-Akt signaling pathway, and chemical carcinogenesis - receptor activation (Supplementary Table S2B, Supplementary Figure S2). Only one common pathway was found between proteome and metabolome data, including glyoxylate and dicarboxylate metabolism (Supplementary Table S2C; Supplementary Figure S3).

**FIGURE 3:**
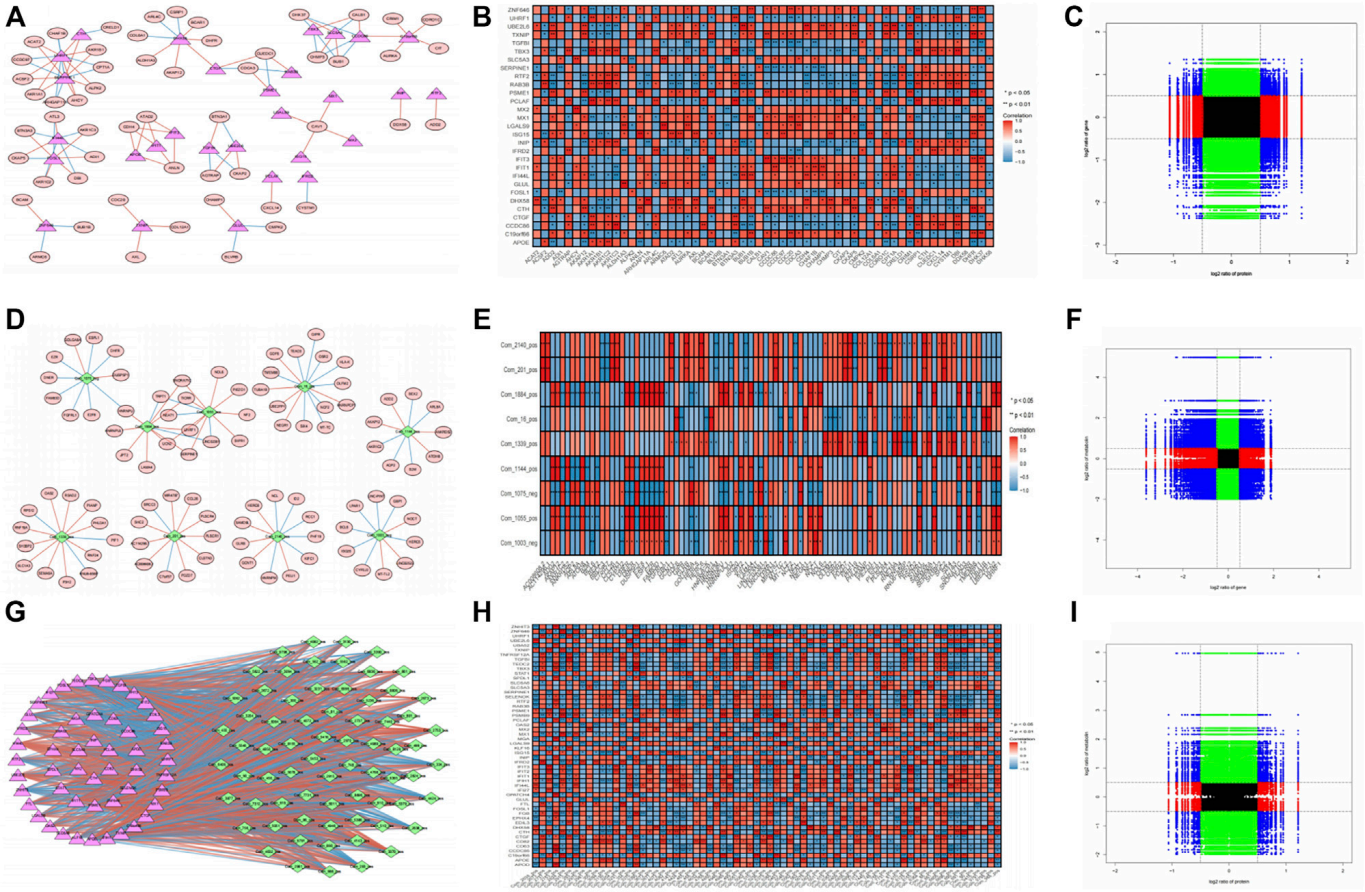
Correlation analysis of transcriptome-proteome, transcriptome-metabolome data, and proteome-metabolome data. **(A)** Interaction network of the DEGs and DEPs with *p*-value <0.05, |cor| > 0.8. Red ellipses represent DEGs, pink triangles represent DEPs, red lines represent positive correlations, and blue lines represent negative correlations. **(B)** Pearson correlation heatmap in transcriptome-proteome analysis. * *p <* 0.05, ** *p <* 0.01; Red indicates positive correlation, and blue indicates negative correlation. **(C)** Nine-quadrant diagram for the transcriptome-proteome correlations. The horizontal axis represents the log2 ratio of protein, and the vertical axis represents the log2 ratio of gene. **(D)** Interaction network of the DEGs and DEMs with *p*-value <0.05, |cor| > 0.8. Red ellipses represent DEGs, green diamonds represent DEMs, red lines represent positive correlations, and blue lines represent negative correlations. **(E)** Pearson correlation heatmap in transcriptome-metabolome analysis. **(F)** Nine-quadrant diagram for the transcriptome-proteome correlations. The horizontal axis represents the log2 ratio of gene, and the vertical axis represents the log2 ratio of metabolin. **(G)** Interaction network of the DEPs and DEMs with *p*-value <0.05, |cor| > 0.8. **(H)** Pearson correlation heatmap in transcriptome-proteome analysis. **(I)** Nine-quadrant diagram for the transcriptome-proteome correlations. The horizontal axis represents the log2 ratio of protein, and the vertical axis represents the log2 ratio of metabolin.

In terms of the correlation analysis, considering the large amount of transcriptome-proteome data, after statistics, 100 correlation pairs were selected from 630 relationship pairs whose correlation was equal to 1 or −1 to construct the network that included 84 nodes (29 proteins and 58 transcriptomes, including three edges, were both proteins and transcriptomes) and 100 edges (Figure 3A). These results were visualized into a heatmap (Figure 3B). The nine-quadrant of the transcriptome-proteome group showed that the genes were highly enriched in the eighth quadrant, followed by the fifth and second quadrants (Figure 3C). Therefore, the majority of proteins showed higher abundances than the relevant RNA in quadrant 8, followed by a relatively large proportion of RNAs and proteins that were commonly expressed with no difference in the fifth quadrant, and a similar proportion of RNAs showed higher abundances than the related proteins in quadrant 2.

In the transcriptome-metabolome combination analysis, 100 correlation pairs were selected from 143 relationship pairs whose correlation is equal to 1 or −1 to construct the network that includes 102 nodes (9 metabolites and 93 transcriptomes) and 100 edges, and the correlation heatmap was plotted (Figures 3D, E). In addition, the nine-quadrant demonstrated that most genes were enriched in the first and seventh quadrants, indicating that the expression abundances of most metabolites were higher than those of the genes (quadrant 1), and the second largest proportion of gene expressed consistently with the metabolic (quadrant 7; Figure 3F).

From the perspective of proteome-metabolome association analysis, a proteome-metabolome network that includes 122 nodes (70 metabolites and 52 proteins) and 100 edges and the correlation heatmap were plotted (Figures 3G, H). Furthermore, the nine-quadrant of proteome-metabolome group showed genes that were highly enriched in the eighth quadrant, followed by the second quadrant (Figure 3I). Therefore, the majority of proteins showed higher abundances than the metabolites in quadrant 8, and a relatively large proportion of metabolites showed higher abundances than the related proteins in quadrant 2.

### 2.4 Six Genes found in common pathways were relevant to GBM prognosis

**FIGURE 4:**
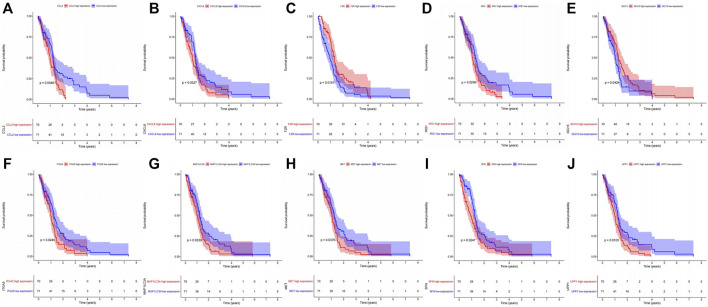
Kaplan–Meier survival analysis between high- and low-expression groups of the 10 genes extracted from the common enriched pathways with significant survival differences in the TCGA dataset. **(A)** CCL2. **(B)** CXCL8. **(C)** F2R. **(D)** IRS1. **(E)** ISG15. **(F)** ITGA5. **(G)** MAP1LC3A. **(H)** MET. **(I)** SFN. **(J)** UPP1.

**FIGURE 5:**
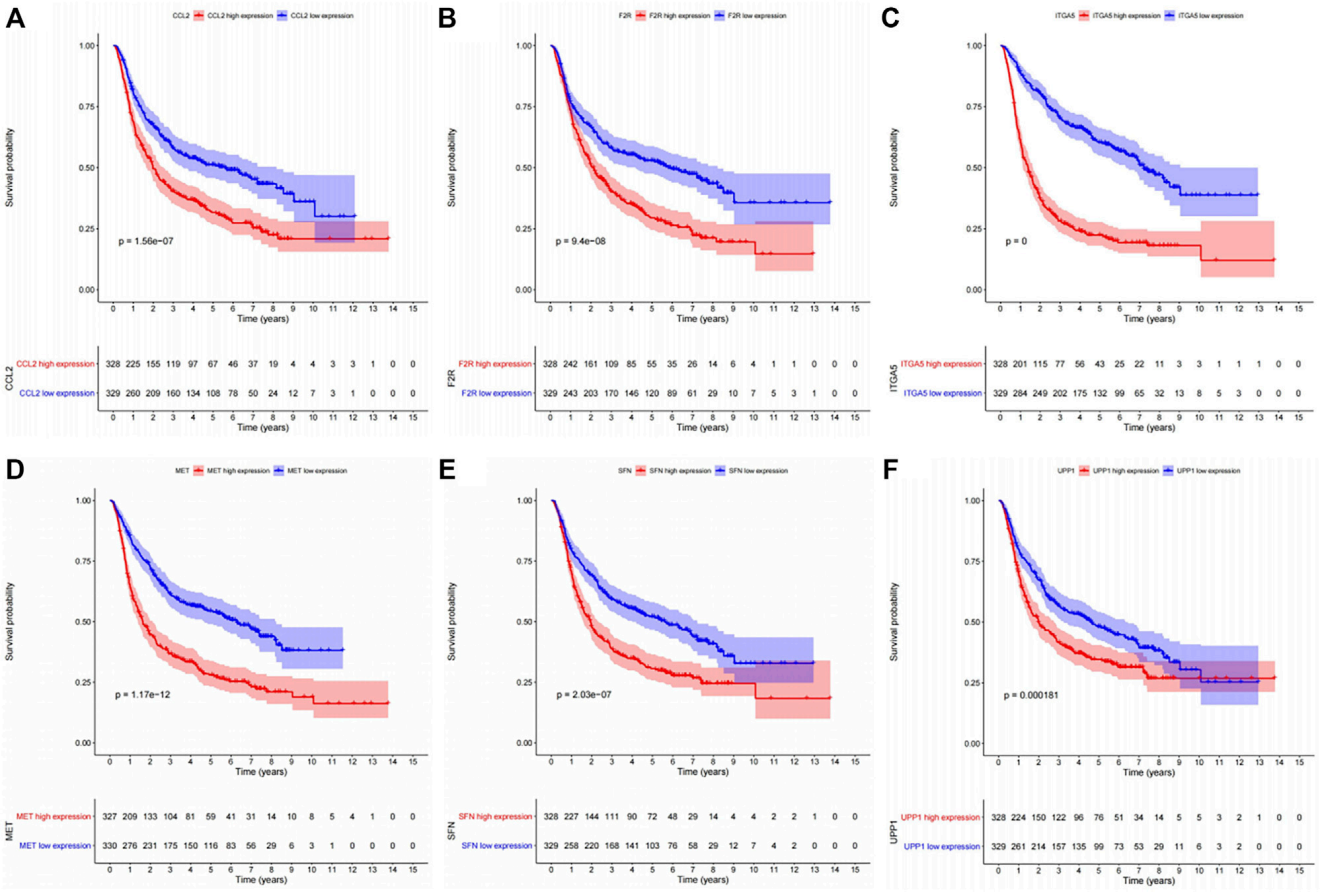
Kaplan–Meier survival analysis between the high- and low-expression groups of the six genes with significant survival differences in the CGGA dataset. **(A)** CCL2. **(B)** F2R. **(C)** ITGA5. **(D)** MET. **(E)** SFN. **(F)** UPP1.

The KEGG enrichment analysis result of transcriptome-proteome-metabolome combination revealed that 11 common pathways were significantly enriched by DEGs, DEPs, and DEMs, including pyrimidine metabolism, FoxO signaling pathway, proteoglycans in cancer, PI3K-Akt signaling pathway, chemical carcinogenesis-ROS, Kaposi sarcoma-associated herpesvirus infection, COVID-19, mitophagy-animal, p53 signaling pathway, chemical carcinogenesis-receptor activation, and ferroptosis (Supplementary Table S3A, Supplementary Figure S4), and 138 genes were found (Supplementary Table S3B). The K-M analysis showed that the survival probabilities of 10 out of 138 genes (CCL2, UPP1, F2R, ITGA5, sulforaphane [SFN], IRS1, CXCL8, MAP1LC3A, MET, and ISG15) were significantly different between the two expression groups in TCGA-GBM (Supplementary Table S3C, Figure 4). Furthermore, 6 out of 10 genes (ITGA5, MET, F2R, CCL2, SFN, and UPP1) in the low-expression group had significantly higher survival probabilities than the high-expression group in CGGA (Supplementary Table S3D, Figure 5).

### 2.5 OXA inhibited the proliferation, migration, and invasion of glioma

**FIGURE 6:**
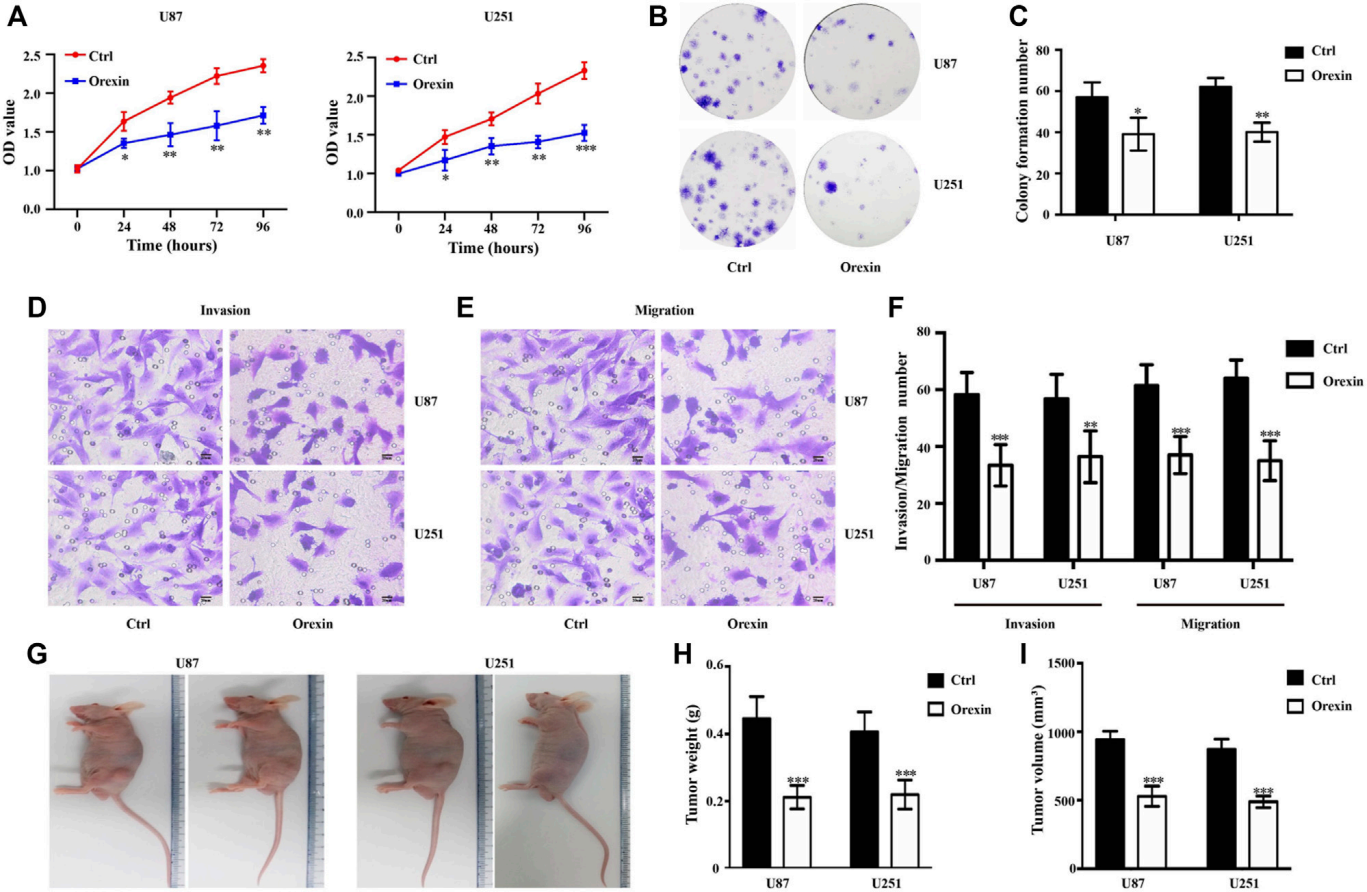
Orexin A (OXA) inhibited the proliferation, migration, and invasion of glioma. **(A)** Effects of OXA treatment on cell proliferation by CCK-8 in U87 and U251 cells. **(B)** Effects and **(C)** statistics of OXA treatment on cell proliferation by colony formation assays in U87 and U251 cells. Effects of OXA treatment on cell migratory **(D)** and invasive **(E)** capacities by transwell assays in U87 and U251 cells, and the statistical information **(F)** of the invasive and migrated glioma cells with or without OXA exposure. **(G)** Observation of the features of tumor weight and volume in subcutaneous xenograft mouse models. **(H)** Tumor weight and **(I)** volume of xenografts derived from U87 and U251 cells. * p < 0.05, ** p < 0.01, *** p < 0.001.

To study the anti-glioma activity of OXA *in vitro*, we performed CCK8 and colony formation assays to investigate cell proliferation. As shown in Figure 6A, the OD value of U87MG and U251 glioma cells was lower in OXA group compared with the vehicle control (0.1% DMSO) group. Meanwhile, the percentages of colony-forming U87MG and U251 glioma cells were reduced after treatment with OXA (Figures 6B, C). Therefore, OXA remarkably impaired the proliferation. Subsequently, the results of transwell assay showed fewer invasive and migrated glioma cells after OXA exposure (Figures 6D–F) The resulting data explained that OXA impaired the migration/invasion of GBM *in vitro*. Moreover, nude mice were subcutaneously xeno-transplanted with U87 or U251 glioma cells *in vivo*. Figures 6G–I show that tumors in nude mice exposure to OXA exhibited significantly lower weight and smaller volume, demonstrating that OXA could effectively inhibit glioma cell growth *in vivo*.

## 3 Discussion

GBM treatment remains extremely challenging, and its prognosis remains unsatisfactory despite surgery, radiotherapy, chemotherapy, and some innovative therapies. Additionally, limited improvement has been realized in the treatment of GBM. OXA inhibits the growth of rat C6 glioma cells through a caspase-dependent mechanism (Biegańska et al., 2012). Programmed cell death has been induced by sustained orexin receptor stimulation in other malignancies (Smart et al., 1999; Ammoun et al., 2006). Similarly, both OXR subtypes can activate cell death as observed in recombinant CHO-S cells (Nicole et al., 2015), acinar cell adenoma (Kaczmarek et al., 2017), colon cancers, and neuroblastoma (Rouet-Benzineb et al., 2004). However, the specific mechanism of orexin’s action has not been fully understood. Detailed exploration of the mechanism of OXA acting on glioblastoma cells could facilitate the understanding of the disease and promote the discovery of therapeutic targets. The transcriptomic, proteomic, and metabolomics data of GBM in the orexin-treated and control groups have been measured, and significant differentially expressed molecules have been identified at each omics level caused by Orexin A, which could well identify whether GBM cells were treated with OXA or not. Further analysis of the correlations of DEGs, DEPs, and DEMs revealed significant correlations among them. Subsequently, the biomolecules and functional pathways, in which OXA might play a role in GBM, have been analyzed.

In our combined transcriptomic-proteome analysis, proteoglycans (PGs) in cancer, chemical carcinogenesis—ROS, and pathways such as mitophagy-animal and p53 signaling pathway were enriched. PGs play important roles in cancer initiation and progression by regulating cellular metabolism, influencing immune surveillance, acting as sensors of mechanical properties, and participating in resistance to various forms of therapy (Wade et al., 2013; Mouw et al., 2014; Baghy et al., 2016). Aberrant ROS plays contradictory roles at different stages of cancer formation, especially in the regulation of cell growth and death, and further understanding of the complex mechanisms of ROS in tumorigenesis is crucial for conquering cancer (Valavanidis et al., 2013; Wang Y. et al., 2021). Huang T. et al. (2021) found that the inhibition of mitophagy partially reversed cannabidiol-induced glioma cell death, suggesting the positive role of mitophagy against tumors. However, FOXO3A-induced mitophagy protects glioma from temozolomide induced cytotoxicity, suggesting that mitophagy could be considered as a double-edge sword for glioma (He et al., 2021; Hu et al., 2021). OXA reduces mitochondrial biogenesis, enhances mitophagy, and damages mitochondrial structure in AD patients (Zhu et al., 2021). OXA may play the same role in tumor and non-tumor diseases. Therefore, it may promote anti-tumor effect by enhancing mitophagy. The P53 tumor suppressor is a key genetic event associated with disease development and progression, and this factor is mutated or absent in 35% of GBM (Ham et al., 2019). P53 signaling pathway plays an important role in the development of glioma. Therefore, OXA might target p53-related pathways to exert its inhibitory effect on cancer. In addition, the majority of proteins showed higher abundances than the relevant RNA in quadrant 8, suggesting post-transcriptional, translational regulation, or accumulation of proteins. Combined transcriptomic-proteome analysis revealed the relationship between transcriptomics and metabolomes, and the FoxO signaling and PI3K-Akt signaling pathway were enriched. Previous mechanistic studies have found that the downregulation of FoxO/Smad signaling promotes cancer cell proliferation in glioblastoma (Wang et al., 2018). The activation of PI3K/Akt/mTOR pathway enhances the proliferation, migration, and invasion of glioma cells, resulting in the occurrence of drug resistance, thereby inhibiting the therapeutic effect of TMZ (Wu et al., 2016; Xu et al., 2018; Zhao et al., 2021). As a result, targeting these pathways by developing corresponding activators or inhibitors may inhibit tumor development and improve patient treatment outcomes. Combined proteomic and metabolome analysis revealed the relationship between the proteome and metabolome, showing only the enrichment of glyoxylate and dicarboxylate metabolic pathways. Glyoxylate and dicarboxylate metabolism plays an important role in prostate cancer (Chen et al., 2021a), but its role in GBM remains to be further investigated. The significant correlation between the differentially expressed molecules in the transcriptomic-proteome-metabolome confirmed the complex regulatory mechanism of OXA acting on GBM. Consequently, further exploration of this regulatory network will help us understand disease processes and identify therapeutic targets.

In the combined transcriptome-proteome-metabolome analysis, 11 common pathways were identified. In addition to PGs described above in cancer, chemical carcinoma-ROS and mitophagy-animal, P53 signaling pathway, also identified pyrimidine metabolism (PyM). A continuous supply of dNTPs is essential for cancer cell survival. Therefore, the permanent activation of the PyM gene is essential for tumor growth (Siddiqui and Ceppi, 2020). OXA may be associated with inhibiting GBM by downregulating the pyrimidine metabolism pathway. In addition, in the FoxO signaling and PI3K-AKT signaling pathway, more DEGs and DEPs are enriched. OXA increases AKT/PDK-1 phosphorylation through phosphatidylinositol 3-kinase and FOXO-1-dependent pathways (Göncz et al., 2008). Ju et al. (2014) revealed that OXA may affect apoptosis in rat hepatocytes by regulating Foxo1 and mTORC1 through the OX1R/PI3K/AKT signaling pathway. Moreover, ferroptosis has attracted our interest, and drugs that target different molecules involved in ferroptosis and stimulating the ferroptosis process are potential adjuvant anticancer treatment options (Liang et al., 2019; Wang et al., 2019; Xu et al., 2019; Chen et al., 2020). In GBM, ferroptosis stimulation can inhibit tumor growth, improve patient survival, and enhance the efficacy of chemoradiotherapy (Yee et al., 2020; Yang et al., 2021). Therefore, OXA targets molecules in the ferroptosis process and activates the ferroptosis pathway to exert anti-tumor effects. These findings need to be further confirmed.

For a good understanding of the mechanism of OXA action, the genes in 11 pathways were extracted, and 138 genes were selected. Survival analysis of TCGA-GBM data was carried out, and 10 genes (CCL2, UPP1, F2R, ITGA5, SFN, IRS1, CXCL8, MAP1LC3A, MET, and ISG15) out of 138 genes had significant survival differences between high- and low-expression groups. The results were further verified in CGGA data, and the results show that six genes (ITGA5, MET, F2R, CCL2, SFN, and UPP1) had significant survival differences between the high- and low-expression groups. The mechanism of action of these genes has been explored. Chen et al. (2021b) reported that ITGA5 accumulation can activate FAK signaling pathway to promote cell growth. Therefore, NEAT1/Mir-128-3p/ITGA5 axis is involved in the occurrence and progression of gliomas. Shi et al. (2021) suggested that ITGA5 expression is upregulated in glioma cells resistant to TMZ, and the overexpression of ITGA5 could increase the resistance of cells to TMZ by promoting the formation of vascular mimicry. OXA downregulated ITGA transcription, thus serving as a pathway for OXA inhibition of glioma. Invasion-related protein ITGA5 in previous studies may be an effective anti-invasion target, which is correlated with advanced tumor grade, recurrence, and overall survival of GBM. [PMID:25853691 and PMID:34873473] (Mallawaaratchy et al., 2015; Wei et al., 2021) Moreover, Wang Q. W et al. (2021) suggested that the MET-STAT4-PD-L1 axis may act with tumor-associated macrophages to enhance immune escape in gliomas and cause poor prognosis in patients with GBM. And Huang et al. (Huang R. et al., 2021) verified that PTPRZ1-MET (ZM) fusion was a key genetic change that drives the progression of low-grade glioma and helped ZM-carrying glioma patients benefit from MET inhibitors. In addition, the inhibition of receptor tyrosine kinases, including MET and/or its ligand hepatocyte growth factor (HGF), is a promising therapeutic strategy against tumor (Suzuki et al., 2010; Ge et al., 2013; Song et al., 2020). Auvergne et al. (Auvergne et al., 2016) strongly demonstrated the importance of the F2R gene encoding PAR1 for the self-renewal and tumorigenicity of glioma A2B5-defined tumor-initiating progenitor cells. CCL2 and its related receptor (CCR2) promote the migration of brain tumors and monocytes across the vascular endothelium (Vakilian et al., 2017; Cho et al., 2019). Besides, Aretz et al. (Aretz et al., 2022) demonstrated that the cross-talk between CCL2 and *β*-catenin could affect the dryness and immune escape mechanism of GBM by regulating the activity of immune cells and glioblastoma stem cells. In animal models, mNOX-E36 blocked angiogenesis and macrophage recruitment, and tumor volume and blood volume were reduced. SFN, which is converted from glucosinolates in broccoli/broccoli buds, prevents chemically induced cancers and inhibits tumor growth in rats (Soundararajan and Kim, 2018; Kaiser et al., 2021; Yan et al., 2021). In tumors, SFN may act by regulating multiple survival signaling pathways by inhibiting carcinogen metabolism, inducing oxidative stress, regulating metabolism, inhibiting cell cycle, and inducing apoptosis (Juengel et al., 2017; Lei et al., 2019; Li et al., 2020; Li et al., 2021). Zhou et al. (Zhou et al., 2020) also revealed the subcellular mechanism by which SFN-CYS (SFN analogue) inhibits human GBM invasion by regulating proteome expression. Methylation of UPP1 was confirmed as a prognostic factor for GBM multiforme in two bioinformatics analyses (Weng and Salazar, 2021; Yu et al., 2021). UPP1 is a potential biomarker of thyroid cancer, and the possible mechanism of regulating epithelial-mesenchymal transition (EMT) plays the role of oncogene (Guan et al., 2019). MethSurv database (Modhukur et al., 2018), a web tool that is used to perform multivariable survival analysis by using DNA methylation data, has been used to perform survival analysis for a CpG located in or around the proximity of a UPP1 in GBM. Results showed multiple methylation modification sites associated with GBM prognosis. Detailed results are shown in Supplementary Figure S5. However, the mechanism of action of these six molecules with OXA is still unclear and requires further investigation.

Our study comprehensively investigated the changes in the transcriptomics, proteomics, and metabolomics levels of GBM cells after OXA treatment, explored the consistency between omics, and screened some possible key pathways, including the FoxO signaling pathway, PI3K-AKT signaling pathway, and ferroptosis. OXA may affect the biological functions of GBM cells by regulating these pathways, and the multi-omics integration tool enhances our ability to focus on specific pathways with potential biological significance in GBM after OXA drug treatment, which will be verified in later studies. In addition, among the 138 genes in 11 pathways screened by multi-omics, six genes (ITGA5, MET, F2R, CCL2, SFN, and UPP1) had independent prognostic roles in both TCGA and CGGA cohorts. Previous *in vitro* and *in vivo* studies have found that ITGA5, MET, F2R, CCL2, and SFN are involved in the regulation of the occurrence, development, invasion, metastasis, and drug resistance of GBM. These processes may be related to GBM inhibition by OXA. Many of these processes still lack validation of biomolecular mechanisms, and the data generated by a large number of omics studies are still underutilized. In addition, the effect of OXA on glioma was determined by performing *in vitro* and *in vivo* experiments. CCK8 and colony formation assays indicated that OXA could inhibit the cell proliferation of glioma cells. Transwell assay results show that OXA impaired the migration/invasion of GBM *in vitro*. Our constructed subcutaneous xenograft mouse models also indicated that OXA could effectively inhibit glioma cell growth.

We acknowledge some limitations of this study. Despite our focus on biologically plausible mechanisms, validation is still lacking, thus requiring further investigation. Additionally, the broad applicability of our conclusions needs to be confirmed. The rigor and credibility of the study results can be improved by integrating multiple data types and mainly reporting the pathways that were jointly found by combining different data types, which were found to be related to the prognostic genes. In conclusion, multi-omics methods were used to effectively understand the interaction mechanism and combined effects of drugs and disease processes.

## 4 Materials and methods

### 4.1 Cell culture

Human GBM cell lines U251 and U87 were obtained from the Chinese Academy of Sciences Cell Bank (Shanghai, China). The cell lines were cultured in DMEM supplemented with 10% fetal bovine serum (FBS, Gibco, United States) and 1% penicillin/streptomycin (Beyotime, China) at 37°C in 5% CO_2_.

### 4.2 Sequencing analysis

The U251 glioma cells were cultured in complete medium with OXA (0.1 μmol/L) for 24 h. Then, total RNA was extracted from two biological repeats of samples in the absence or presence of OXA. The total amount and integrity of RNA were assessed using the RNA Nano 6000 assay kit of the Bioanalyzer 2,100 system (Agilent Technologies, CA, United States). The cDNA fragments with length of 370–420 were selected by purifying the library fragments with the AMPure XP system (Beckman Coulter, Beverly, United States). Then, by using PCR amplification, the PCR product was purified by AMPure XP beads, and the library was finally obtained. After the library was qualified, the different libraries were pooled according to the effective concentration and the target amount of data off the machine, and then being sequenced using Illumina NovaSeq 6,000.

After being digested with trypsin, the proteins were extracted and labelled with multiplexed tandem mass tag (TMT) reagents. The intensity of TMT reporter ions was extracted using mobile phase A (2% acetonitrile, pH was adjusted to 10.0 by using ammonium hydroxide) and B (98% acetonitrile). For transition library construction, shotgun proteomics analyses were performed using an EASY-nLCTM 1200 UHPLC system (Thermo Fisher) coupled with a Q ExactiveTM HF-X mass spectrometer (Thermo Fisher) operating in the data-dependent acquisition (DDA) mode. The quality of analysis results was improved using the PD 2.4 software further to filter the retrieval results: Peptide Spectrum Matches (PSMs) with a credibility of more than 99% was identified PSMs.

Untargeted LC-MS/MS analyses were performed using an Vanquish UHPLC system (ThermoFisher, Germany) coupled with an Orbitrap Q ExactiveTM HF mass spectrometer (Thermo Fisher, Germany) in Novogene Co., Ltd. (Beijing, China) in both positive and negative modes. The spray voltages of positive and negative ionization modes were both 3.5 kV. The raw data files generated by UHPLC-MS/MS were processed using the Compound Discoverer 3.1 (CD3.1, ThermoFisher) to perform peak alignment, peak picking, and quantitation for each metabolite.

### 4.3 Data sources

The transcriptome, proteomic, and metabolome data of three repeats of GBM cells treated with OXA and three repeats of GBM cells without treatment were acquired from sequencing analysis. The transcriptome and proteomic contained 57,169 and 6,020 data, respectively. In terms of the metabolome data, 481 metabolites were detected in positive mode, and 196 metabolites were detected in negative mode.

### 4.4 Screening of DEGs, DEPs and DEMs

**FIGURE 7:**
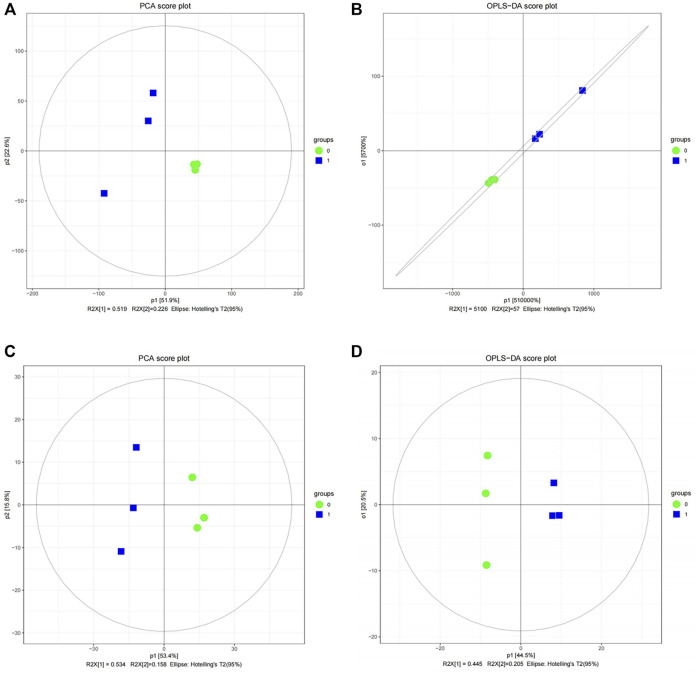
Quality analysis of proteomic and metabolome data. **(A)** Principal component analysis (PCA) score diagram for the differential expression analysis of proteomic data between case and control samples. **(B)** Orthogonal partial least-squares-discriminant analysis (OPLS-DA) score diagram for the differential expression analysis of proteomic data between case and control samples. **(C)** PCA score diagram for the differential expression analysis of metabolome data between case and control samples. **(D)** OPLS-DA score diagram for the differential expression analysis of metabolome data between case and control samples.

The limma package (version 3.44.3) was applied to 57,169 transcriptome data directly to screen DEGs between case and control groups with threshold values of |log2fold change (FC)| > 0.5 and *p*-value <0.05. In terms of the differential expression analysis of proteomic data, principal component analysis (PCA) was first performed to analyze the aggregation and dispersion of the six samples. Then, orthogonal partial least squares discriminant analysis (OPLS-DA) was employed to the six samples to calculate the variable important in projection (VIP) value of each sample. Considering that the PCA and OPLS-DA results suggested that normal and case samples could be well separated (Figures 7A, B), differential analysis was performed on the proteome data by using limma. The DEPs were screened out with |log_2_FC| > 0.5, *p*-value < 0.05, and VIP > 1.

From the perspective of DEMs, PCA and OPLS-DA were initially used for the 481 metabolites in the positive mode and 196 metabolites in the negative mode, respectively. The PCA and OPLS-DA results of the two modes suggest that normal and case samples could be well separated (Figures 7C, D). Therefore, limma can be directly applied to metabolome data in positive and negative modes to select DEMs in each mode at threshold values of |log2FC| > 0.5, *p*-value < 0.05, and VIP > 1.

### 4.5 Functional enrichment analyses of DEGs, DEPs, and DEMs

ClusterProfiler (version 3.16.0) was employed to conduct GO and KEGG enrichment analyses on DEGs with screening criteria of *p <* 0.05. Simultaneously, the functional enrichment analyses of DEPs were performed in KEGG and REACTOME databases. Furthermore, the expression levels of DEMs were extracted and used as input into MetaboAnalyst (https://www.metaboanalyst.ca/), which includes the KEGG, HMDB, and STITCH databases, to perform enrichment analyses. Next, a PPI network was plotted to determine the interaction relationship among DEPs through STRING website (https://string-db.org) with the setting of confidence = 0.15, and the results were visualized using Cytoscape (version 3.7.2).

### 4.6 Transcriptome-proteome, transcriptome-metabolome, proteome-metabolome association analyses

The interactions between transcriptome-proteome, transcriptome-metabolome, and proteome-metabolome was determined by applying combined KEGG analysis to each combined group. The DEGs-DEPs, DEGs-DEMs, and DEPs-DEMs were used as input into KEGG enrichment analysis, and the common enriched pathways in transcriptome-proteome, transcriptome-metabolome, and proteome-metabolome groups were tested by hypergeometric analysis.

Correlation analysis was further examined by calculating the Pearson correlation coefficient in each group (transcriptome-proteome, transcriptome-metabolome, and proteome-metabolome) at threshold values of *p*-value <0.05 and |cor| > 0.8. Then, the log2FC value of each selected items in each group was further computed and visualized into nine-quadrant by using the omicshare platform (http://www.omicshare.com/tools/Home/Soft/jxx). A total of 57,169 transcriptome and 6,020 proteome data in transcriptome-proteome, 57,169 transcriptome and 677 metabolome data in transcriptome-metabolome, and 6,020 proteome and 677 metabolome data in proteome-metabolome were recorded.

### 4.7 Transcriptome-proteome-metabolome association analysis

The transcriptome-proteome-metabolome interactions were investigated using all the data in the three groups as input into the combined KEGG analysis. All common enriched pathways of DEGs, DEPs, and DEMs were tested by hypergeometric distribution test. Then, the genes in the common enriched pathways were extracted, and their survival situations were further analyzed in TCGA-GBM data set by Kaplan-Meier (K-M) survival analysis. The genes with significant survival differences between high- and low-expression groups were selected. Then, the survival probabilities of these selected genes between different expression groups were subsequently analyzed in CGGA database by K-M analysis at a statistical significant threshold of *p* < 0.05.

### 4.8 Colony formation assay

U251 or U87 glioma cells were cultured in six-well plates at a density of 500 cells/well. Cells were suspended in DMEM supplemented with 10% FBS for 24 h. Then, the glioma cells were cultured in complete medium with OXA (0.1 μmol/L) for 1 week to allow colony formation. Cells were fixed with 4% paraformaldehyde and stained with 0.1% crystal violet. The number of clones (>50 cells) was counted under the microscope.

### 4.9 Cell counting kit 8 (CCK8)

For cell proliferation assay, U251 or U87 glioma cells were planted into a 96-well plate at a concentration of 5,000 cells/well and treated with medium OXA (0.1 μmol/L) for 24, 48, 72, and 96 h. Then, 10 µL of CCK8 (Beyotime) was added to each well, and the cells were incubated for 1 h at 37°C. The absorbance values were read at 450 nm by using an enzyme-linked instrument.

### 4.10 Transwell migration and invasion assay

In migration assay, the suspension containing 1×10^5^ glioma cells with serum-free DMEM media was placed in the upper chamber of a Transwell insert (Corning, United States), and 500 µL of complete medium was added into the bottom chambers. In the invasion assay, Matrigel (BD, United States) was coated on the upper chambers seeded with 2 × 10^5^ cells, and the lower chamber contained 500 µL of complete medium. Then, OXA (0.1umol/L) was added into the upper chamber, and Transwells were incubated for 36 h at 37°C. Then, cells were fixed with 4% paraformaldehyde and stained with 0.1% crystal violet for 15 min. Migrated/invasive glioma cells were photographed under a microscope.

### 4.11 Mouse xenografts

All animal experiments were approved by the Institutional Animal Care and Use Committee of Guizhou Provincial People’s Hospital. U251 or U87 glioma cells were re-suspended in DMEM and were injected subcutaneously into nude mice. OXA (0.1 g/kg) was injected into mice intraperitoneally daily for 2 weeks starting 1 week after tumor implantation, while sterile double-steamed water with the same dose was injected into the control group. The mice were immolated at predetermined times and tumor volume and weight were recorded.

### 4.12 Statistical analysis

R software (https://www.r-project.org/) was used in the current study. All data are expressed as the mean ± SD. The differences between groups were analyzed by one-way analysis of variance, followed by Tukey’s *post hoc* test. The relationship between patient survival and genes expression was tested with the log-rank test and plotted with the Kaplan–Meier curves. If not specified above, *p*-value less than 0.05 was considered statistically significant.

## Data Availability

The datasets presented in this study can be found in online repositories. The names of the repository/repositories and accession number(s) can be found in the article/Supplementary Material.

## Supplementary material

SUPPLEMENTARY FIGURE S1 KEGG enrichment analysis in the combined analysis of transcriptome-proteome data. Six common pathways were identified, including p53 signaling pathway **(A)**, mitophagy-animal **(B)**, Kaposi sarcoma-associated herpesvirus infection **(C)**, coronavirus disease **(D)**, Proteoglycans in cancer **(E)**, and chemical carcinogenesis - reactive oxygen species **(F)**.

SUPPLEMENTARY FIGURE S2 KEGG enrichment analysis in the combined analysis of transcriptome-metabolome data. Four common pathways were identified, including pyrimidine metabolism **(A)**, FoxO signaling pathway **(B)**, PI3K-Akt signaling pathway **(C)**, and chemical carcinogenesis—receptor activation **(D)**.

SUPPLEMENTARY FIGURES S3 KEGG enrichment analysis in the combined analysis of proteome-metabolome data. Glyoxylate and dicarboxylate metabolism was identified as common pathways.

SUPPLEMENTARY FIGURE S4 KEGG enrichment analysis in the combined analysis of transcriptome-proteome-metabolome. Eleven common pathways were identified, including pyrimidine metabolism **(A)**, PI3K-Akt signaling pathway **(B)**, ferroptosis **(C)**, mitophagy-animal **(D)**, Kaposi sarcoma-associated herpesvirus infection **(E)**, proteoglycans in cancer **(F)**, FoxO signaling pathway **(G)**, Coronavirus disease **(H)**, chemical carcinogenesis-reactive oxygen species **(I)**, chemical carcinogenesis-receptor activation **(J)**, and p53 signaling pathway **(K)**.

SUPPLEMENTARY FIGURES S5
**(A)** Heat maps show the results of cluster analysis of individual CPGS in the UPP1 gene, correlating methylation levels with available patient characteristics and gene subregions. Methylation level (1 = complete methylation; 0 = completely unmethylated) is shown as a continuous variable from blue to red. The rows correspond to CPGS, and the columns correspond to patients. **(B–K)** Kaplan–Meier survival analysis between high- and low-UPP1 methylation groups of glioblastoma multiforme.

SUPPLEMENTARY TABLE S1A A total of 806 GO terms were enriched by DEGs between case and control samples.

SUPPLEMENTARY TABLE S1B Eleven KEGG pathways were enriched by DEGs between case and control samples.

SUPPLEMENTARY TABLE S1C A total of five KEGG pathways were enriched by DEPs between case and control samples.

SUPPLEMENTARY TABLE S1D A total of 136 REACTOME pathways were enriched by DEPs between case and control samples.

SUPPLEMENTARY TABLE S1E Fourteen pathways were enriched by DEMs between case and control samples.

SUPPLEMENTARY TABLE S2A Six common pathways were identified between Transcriptome and Proteome data.

SUPPLEMENTARY TABLE S2B Four common pathways were identified between transcriptome and metabolome data.

SUPPLEMENTARY TABLE S2C Glyoxylate and dicarboxylate metabolism was identified as common pathway between proteome and metabolome data.

SUPPLEMENTARY TABLE S3A Eleven common KEGG pathways were significantly enriched by DEGs, DEPs, and DEMs in transcriptome-proteome-Metabolome combination data.

SUPPLEMENTARY TABLE S3B The 138 genes extracted from the 11 common KEGG pathways.

SUPPLEMENTARY TABLE S3C Ten out of 138 genes were significantly associated with GBM survival in TCGA-GBM cohort.

SUPPLEMENTARY TABLE S3D Six out of 138 genes were significantly associated with GBM survival in CGGA cohort.
