# Supplementary figures and images for: Multiomics integration reveals the effect of Orexin A on glioblastoma
Source: Front Pharmacol. 2023 Jan 20;14:1096159. doi: 10.3389/fphar.2023.1096159 (PMC9894894; doi:10.3389/fphar.2023.1096159)

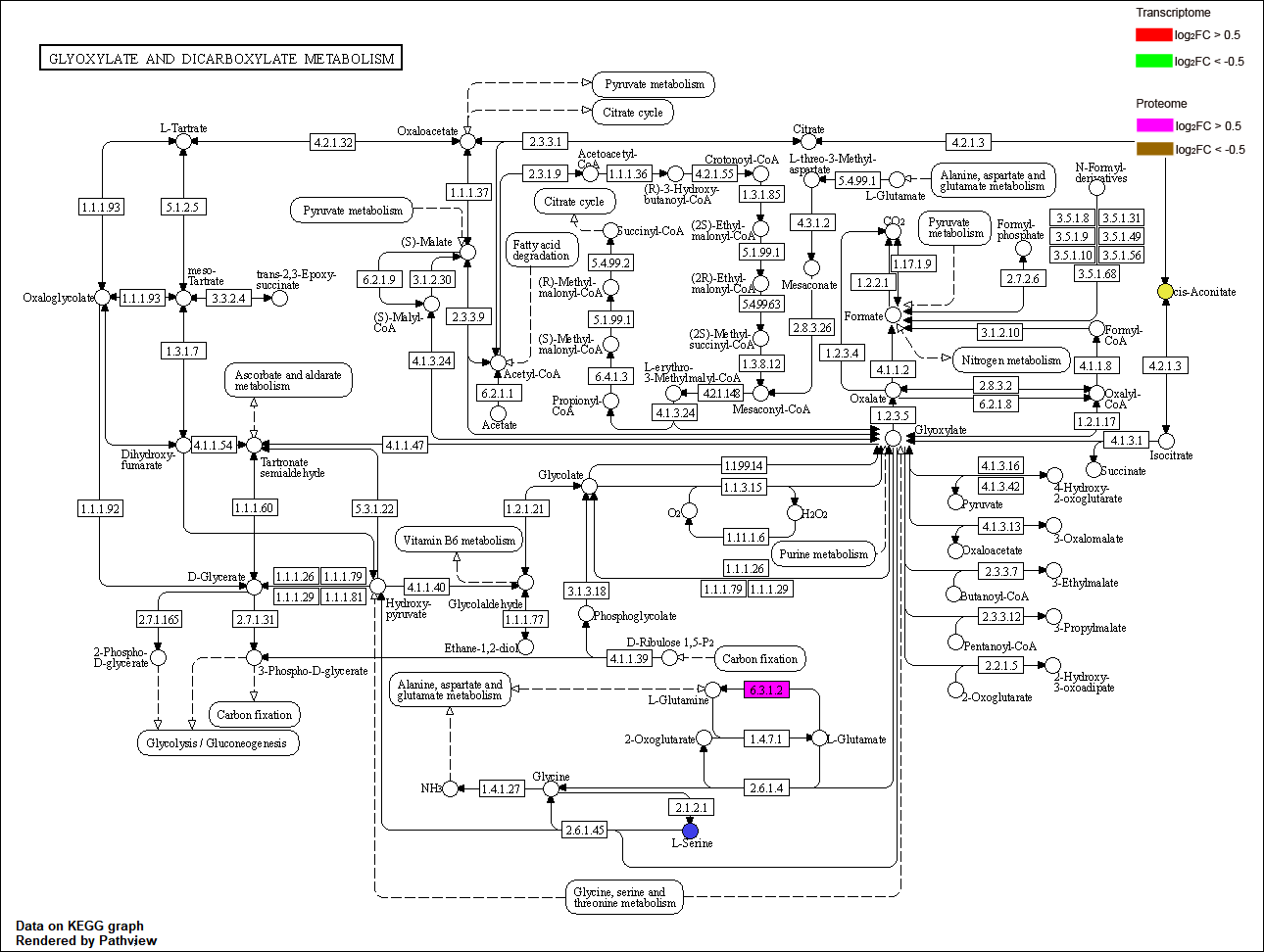

Supplement: Supplementary file 1 [file Image3.TIF]

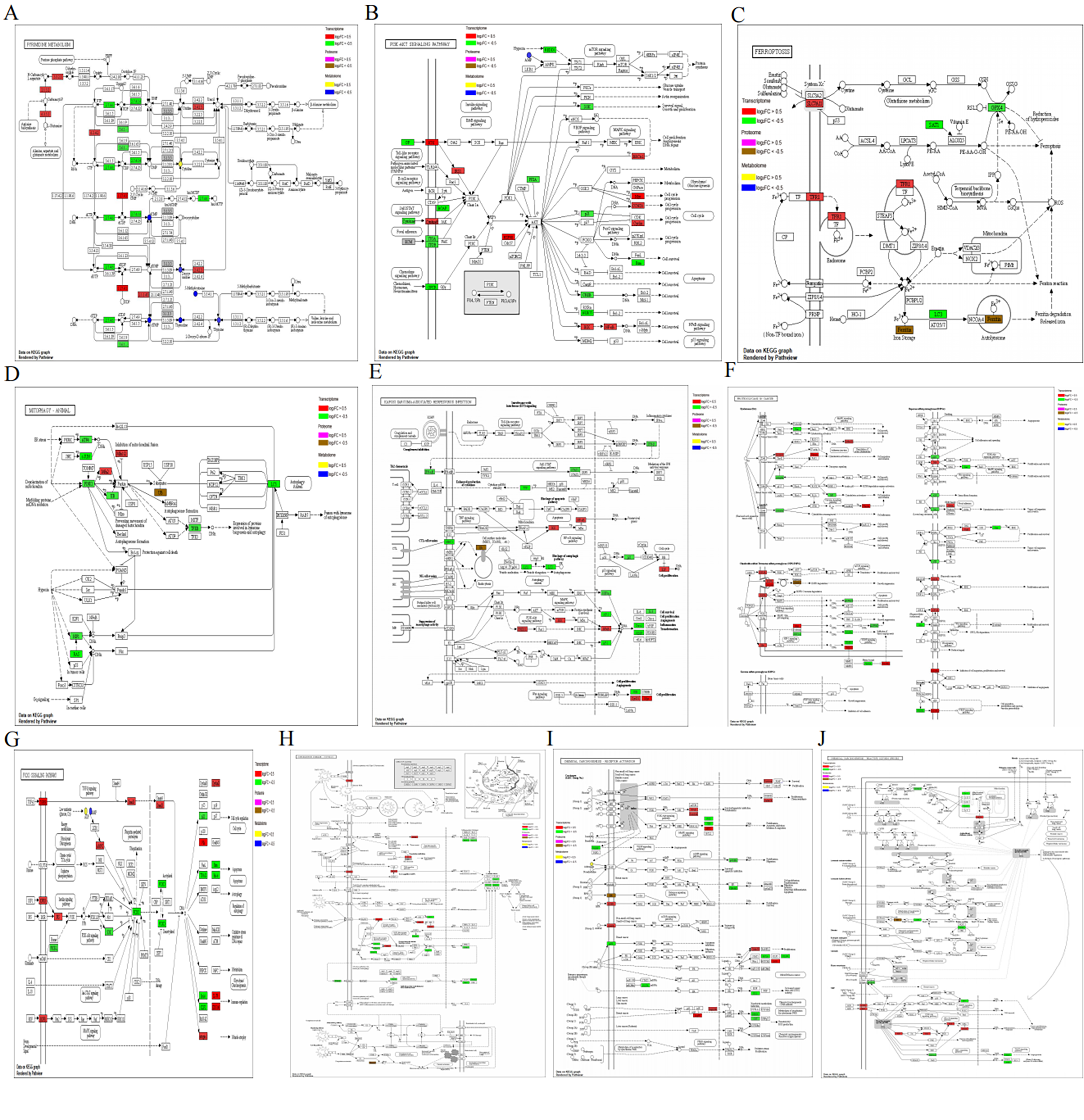

Supplement: Supplementary file 2 [file Image4.TIF]

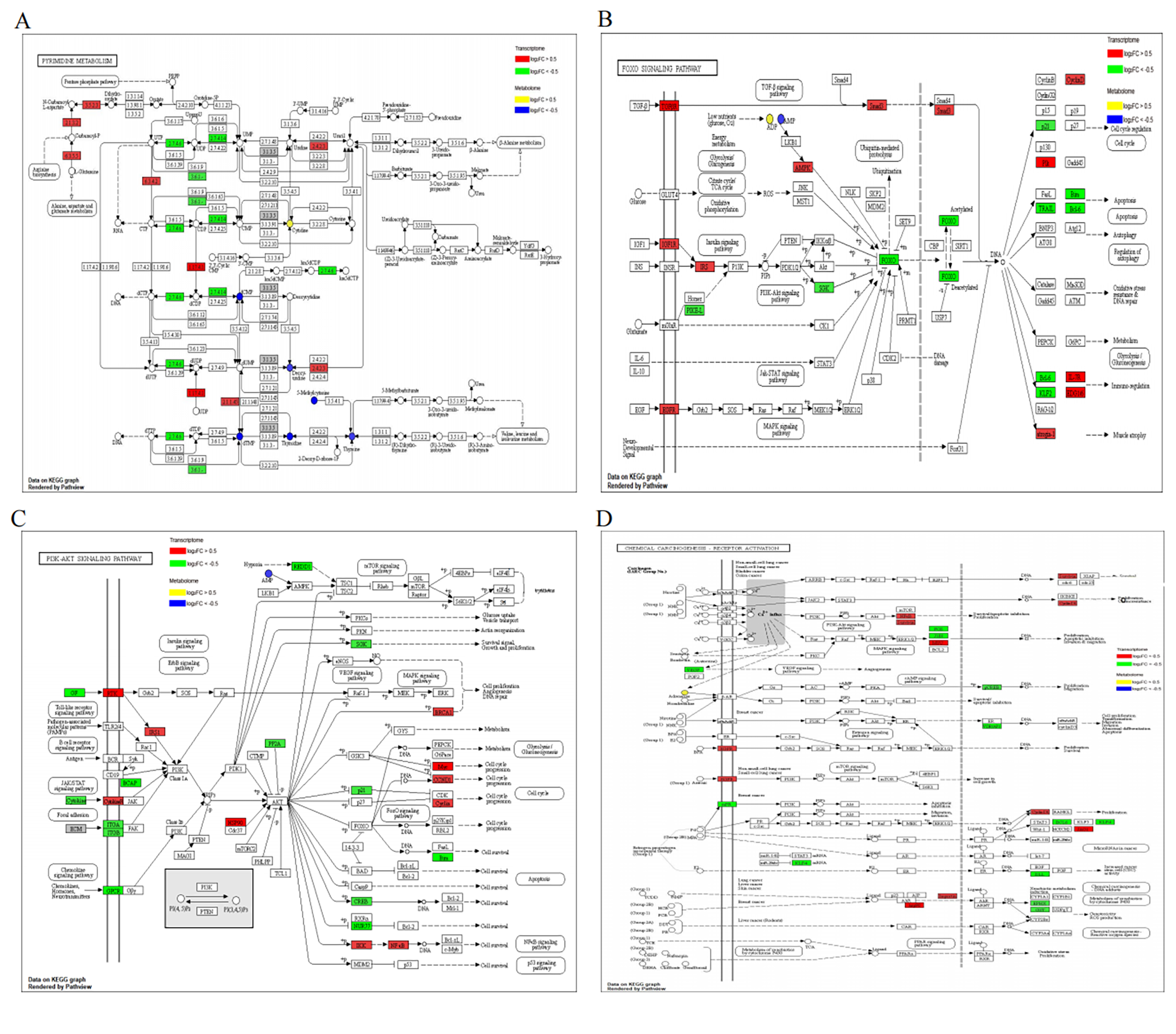

Supplement: Supplementary file 4 [file Image2.TIF]

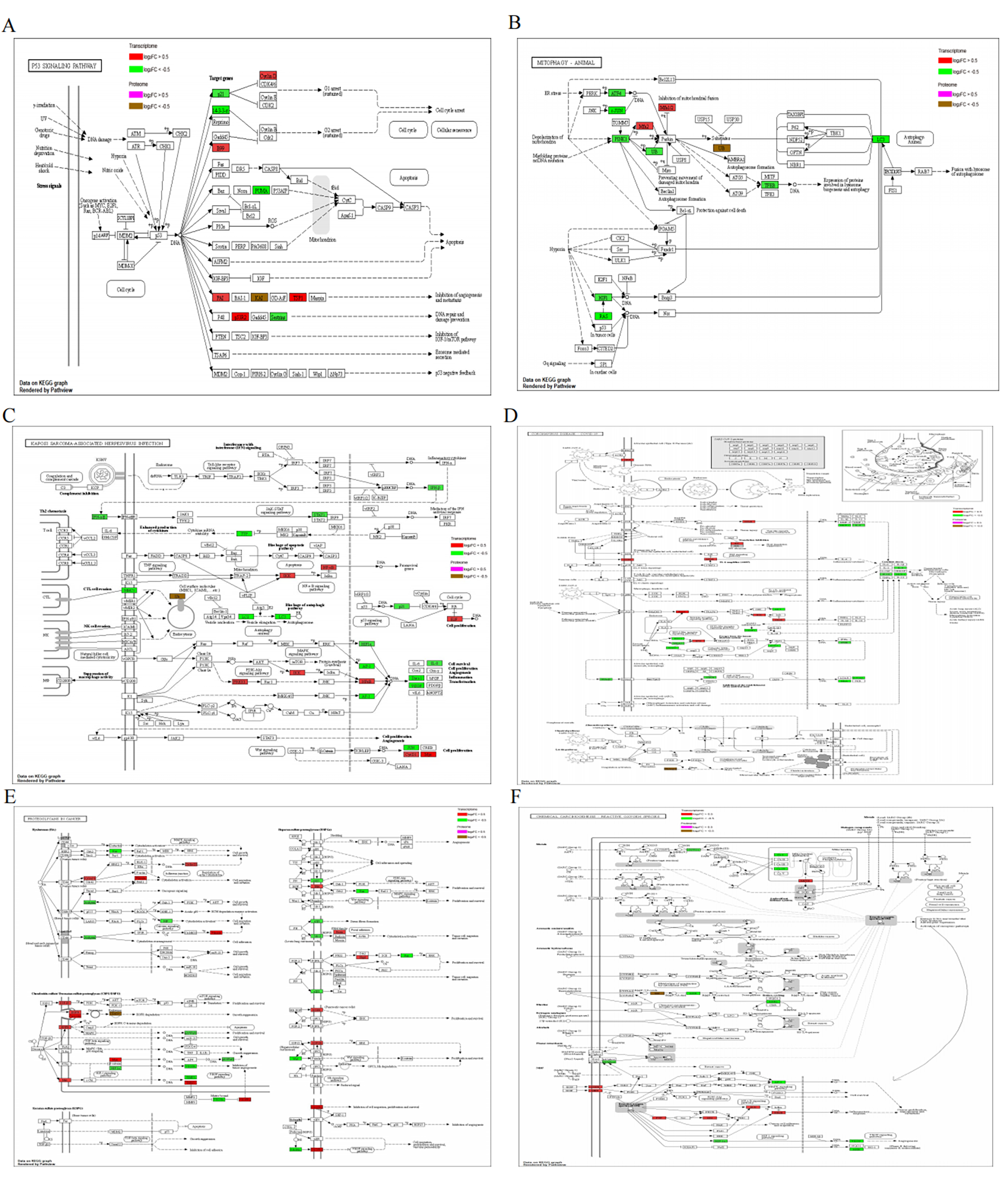

Supplement: Supplementary file 5 [file Image1.TIF]

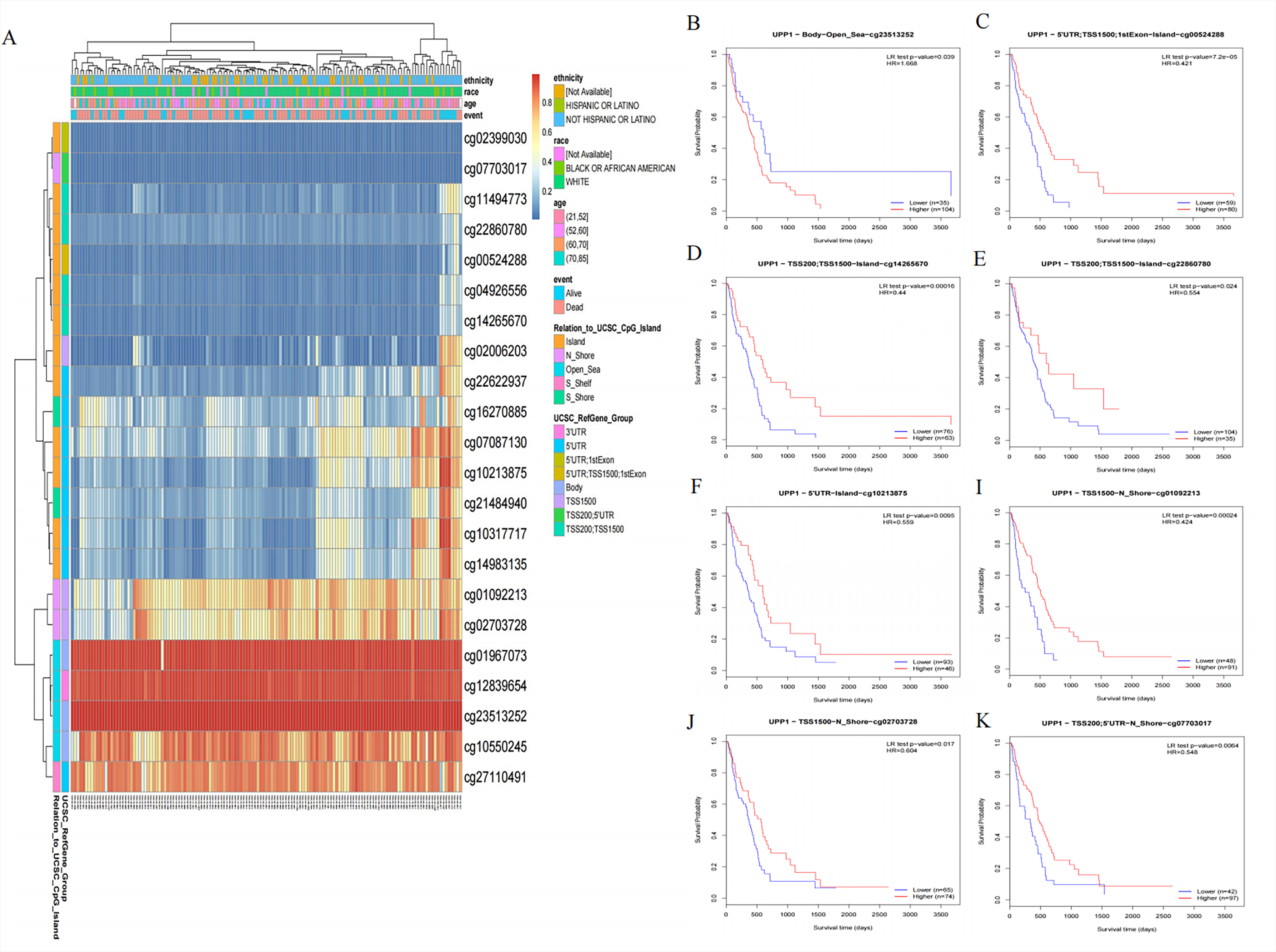

Supplement: Supplementary file 6 [file Image5.TIF]
